# An Account of Diseases in an Eastern District of London from March 20, to April 20, 1809

**Published:** 1809-05

**Authors:** 


### *An Account of Diseases in an Eastern District of London from March 20, to April 20, 1809.*

| | |
|---|---|
| ACUTE DISEASES. | |
| Pneumonia | 3 |
| Peripneumonia Notha | 8 |
| Pleuritis | 2 |
| Cynanche Tonsillaris | 3 |
| Rheumatismus Acutus | 4 |
| CHRONIC DISEASES. | |
| Dyspnœa | 11 |
| Tussis cum Dyspnœa | 20 |
| Tussis | 12 |
| Phthisis Pulmonalis | 3 |
| Pleurodyne | 4 |
| Palpitatio | 3 |
| Enterodynia | 2 |
| Cephalalgia | 4 |
| Vertigo | 3 |
| Hysteria | 2 |
| Hypochondriasis | 2 |
| Fluor Albus | 5 |
| Amenorrhœa | 4 |
| Menorrhagia | 2 |
| Rheumatismus Chronicus | 12 |
| PUERPERAL DISEASES. | |
| Dolor post Partum | 4 |
| Dysuria | 3 |
| Diarrhœa | 3 |
| Rhagas Papillæ | 4 |
| Mastodynia | 4 |
| INFANTILE DISEASES. | |
| Diarrhœa | 5 |
| Vermes | 3 |
| Dentitio | 4 |
| Pertussis | 2 |
| Tinea | 1 |

The continuance of severely cold weather, which has been protracted to a considerable length, with very short intervals of a different temperature, together with the prevalence valence of easterly winds, have produced unpleasant effects on the human frame. Thunder and lightening, and a pretty considerable fall of snow, occurring within a few days of each other, is an unusual phenomenon. Such sudden varieties are adapted to occasion as sudden alterations in the animal system, and particularly to affect those who are labouring under diseases of the lungs. We have, therefore, to announce a continuance and increase of the same complaints of the pulmonary system which formed a principal figure in the last Report.

